# Cytokine expression profile in hamsters immunized with OmpL37 from *Leptospirainterrogans *in different vaccine formulations

**DOI:** 10.1186/1753-6561-8-S4-P164

**Published:** 2014-10-01

**Authors:** Thaís Oliveira, André Grassmann, Rodrigo Schuch, Mariana Pereira, Daiane Hartwig, Alan McBride, Odir Dellagostin

**Affiliations:** 1 Laboratório de Vacinologia, Unidade de Biotecnologia, CDTec, Universidade Federal de Pelotas, Pelotas, RS, Brazil; 2 Laboratório de Pesquisa em Doenças Infecciosas, Unidade de Biotecnologia, CDTec, Universidade Federal de Pelotas, Pelotas, RS, Brazil; 3 Departamento de Microbiologia e Parasitologia, Instituto de Biologia, Universidade Federal de Pelotas, Pelotas, RS, Brazil

## Background

Pathogenic spirochetes from the genus *Leptospira *are the bacteria that cause leptospirosis, an emerging zoonosis responsible for over 500,000 human cases each year [1]. Vaccination with inactivated whole-cell preparations (bacterins) has limited efficacy due to the wide antigenic variation of the pathogen. Bacterins are reactogenic and confers serovar specific and short-term immunity [2]. The protein OmpL37 represents a potential target for vaccine development against leptospirosis since it is recognized by human and animal serum, binds human extracellular matrix components, is up-regulated in vivo and conserved among pathogenic leptospires [3]. We aimed to evaluate the immune response induced by OmpL37 from *L. interrogans *serovar Copenhageni strain Fiocruz L1-130 in hamsters, using prime-boost, DNA, and protein-based immunizations.

## Methods

The *ompL37 *gene was cloned into pAE and pTargeT vectors, to obtain a subunit and a DNA vaccine, respectively. The recombinant protein OmpL37 (rOmpL37) was characterized by Western blot (WB) and pTargeT/*ompL37 *was evaluated by transfection of CHO-K1 cells and analyzed by immunofluorescence. Groups of 6 hamsters were immunized twice with an interval of 21 days as follows: rOmpL37-Alhydrogel (2x 100 µg), pTargeT-*ompL37 *(2x 100 µg), prime-boost pTargeT-*ompL37 *(100 µg) plus rOmpL37 (100 µg), pTargeT (2x 100 µg) and PBS-Alhydrogel. Two independent experiments were conducted. Pooled blood samples, collected at days 0, 21 and 42, were processed for RNA isolation using the RiboPure-Blood Kit (Ambion). cDNA synthesis was performed using the High-Capacity cDNA Reverse Transcription Kits (Applied Biosystems). Expression profiles of IFN-γ, TNF-α, IL1-α and TGF-β were accessed by quantitative real-time PCR using SYBR Green PCR Master Mix (Applied Biosystems). The relative Ct (ΔΔC_T_) method was used to quantify cytokine gene expression. The CT of each test gene was evaluated in pooled hamster whole-blood samples, the CTs were normalized against the *β-actin *gene CT (ΔCT) and then compared to the same normalized gene in the respective control groups (calibrator) [4].

## Results and conclusion

Considering that target genes are up or down-regulated when a 2-fold change in mRNA levels is observed [5], TNF-α was induced by rOmpL37 at day 42 (ratio = 2.84), and by pTargeT/*ompL37 *at days 21 and 42 (ratio > 5). In contrast, IFN-γ was down regulated in the prime-boost group at day 42 (ratio = 0.41). Similarly, down-regulation of IL1-α was observed at day 42 in the pTargeT/*ompL37 *(ratio = 0.28) and prime-boost (ratio = 0.19) groups. TGF-β was expressed at basal levels in all groups. Both rOmpL37 and pTargeT/*ompL37 *were able to induce a pro-inflammatory response, characterized by increased TNF-α expression. However, the Th1 and pro-inflammatory cytokine levels decreased in the prime-boost group.
